# Validity of estimating center of pressure during walking and running with plantar load from a three-sensor wireless insole

**DOI:** 10.1017/wtc.2022.5

**Published:** 2022-06-06

**Authors:** Richard A. Brindle, Chris M. Bleakley, Jeffrey B. Taylor, Robin M. Queen, Kevin R. Ford

**Affiliations:** 1 Department of Physical Therapy, High Point University, High Point, North Carolina, USA; 2 Baylor University – Keller Army Community Hospital Division 1 Sports Physical Therapy Fellowship, United States Military Academy, West Point, New York, USA; 3 School of Health Sciences, Faculty of Life and Health Sciences, Ulster University, Newtownabbey, United Kingdom; 4 Department of Biomedical Engineering and Mechanics, Virginia Tech, Blacksburg, Virginia, USA

**Keywords:** centre of pressure, plantar force, three sensor insole, validity

## Abstract

The purpose of this study was to determine if estimated center of pressure (COP) from plantar force data collected using three-sensor loadsol insoles was comparable to the COP from plantar pressure data collected using pedar insoles during walking and running. Ten healthy adults walked and ran at self-selected speeds on a treadmill while wearing both a loadsol and pedar insole in their right shoe. Plantar force recorded from the loadsol was used to estimate COP along mediolateral (COPx) and anteroposterior (COPy) axes. The estimated COPx and COPy were compared with the COPx and COPy from pedar using limits of agreement and Spearman’s rank correlation. There were significant relationships and agreement within 5 mm in COPx and 20 mm in COPy between loadsol and pedar at 20–40% of stance during walking and running. However, loadsol demonstrated biases of 7 mm in COPx and 10 mm in COPy compared to pedar near initial contact and toe-off.

## Introduction

Loading magnitude and distribution may be two important risk factors for lower extremity overuse injuries (Bertelsen et al., 2017). In the absence of laboratory-grade equipment, magnitude and distribution of load can be objectively quantified with force- and pressure-sensing shoe insoles (Seiberl et al., 2018; Burns et al., 2019; Renner et al., 2019). The loadsol (novel GmbH, Munich, Germany) is a wireless force-sensing insole that records data from up to three sensors per insole on mobile devices using the loadsol application. Plantar pressure insoles, like the pedar system (novel GmbH, Munich, Germany), have up to 99 sensors per insole and require additional equipment and cabling for sufficient power and data storage, which may limit their use in clinical or outside of the laboratory settings (in-field). Alternatively, the loadsol is well suited for collecting data in the clinic and in-field (Peebles et al., 2018, 2019). Plantar pressure insoles quantify both the magnitude and distribution of loads, while loadsol quantifies loading magnitudes only. However, loadsol insoles that contain at least three force sensors may be capable of estimating center of pressure (COP), thus providing an estimate of both loading magnitude and distribution in settings where plantar pressure insoles may not be feasible. Thus, the purpose of this study was to determine if estimated COP from three-sensor loadsol was similar to the COP from plantar pressure insoles during walking and running.

## Materials and Methods

### Participants

Prior to participant recruitment, this protocol was approved by High Point University’s Institutional Review Board. Healthy adults aged 18–60 years old who wore either a size (UK) 5.5 or 9.5 shoes were recruited to participate. All participants gave their written informed consent prior to data collection. Ten healthy adults (six men, four women; 31 ± 9 years; 1.73 ± 0.07 m; 75.8 ± 15.4 kg) participated in this study.

### Procedures

For data collection, participants changed into athletic shorts and their height and weight were measured. All participants wore standard laboratory shoes (Adidas adiPURE, Portland, OR). Pedar and loadsol sensors are shaped as shoe insoles and come in a variety of sizes, which are meant to fit inside a shoe during use. The factory insole of the right shoe was removed and a right-side pedar and three-sensor loadsol insole were stacked on top of one another in the shoe. Stacking arrangement of the insoles was randomized between participants, and the insoles were calibrated following manufacturer instructions. Self-selected treadmill speed was determined for both walking and running following a previous protocol (Ford et al., 2013) before walking and running trial was recorded in a laboratory setting. For each trial, participants were instructed to make a hard stomp with their right foot and then walk or run for 1 min. The stomp created a unique signal with a well-defined peak that was used for time synchronizing pedar and loadsol data in postprocessing. Procedures were then repeated with insoles stacked in the reverse-randomized arrangement.

### Data Analysis

The three-sensor loadsol had sensors under the heel, medial forefoot, and lateral forefoot. Force data from loadsol were recorded at the maximum sampling frequency of 200 Hz using the loadsol application on a mobile device. Force and COP data from pedar were also recorded at 200 Hz, the maximum sampling frequency when only using one insole, and stored in Novel Database Pro software (novel GmbH, Munich, Germany) on a laptop. Custom Matlab software (MathWorks, Natick, MA) processed trial data. Trial data from loadsol and pedar were synchronized based on the time of peak force from the stomp that preceded each movement trial. Stance phases were determined using 50 N thresholds for initial contact and toe-off events. COP and force data were filtered with a dual-pass fourth-order Butterworth filter at 20 Hz for walking data, and 50 Hz for running data. Filter frequencies were determined based on residual analyses from data recorded on the first five participants who enrolled in this study (Winter, 2009). Force data from the three loadsol sensors were entered into equations to estimate COP along the mediolateral (COPx) and anteroposterior (COPy) axes (Equations (1) and (2)). Precision of the estimated COP from the loadsol is limited by the number of sensors. Thus, these equations to estimate COP using loadsol data assume that pressure was centered over the sensor’s midpoint ( 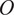 ) in instances when total force was recorded by only one sensor. Time-series COPx and COPy for the pedar and loadsol were time normalized to 201 data points and 101 data points for walking and running, respectively. Ensemble averages of COPx and COPy were generated for each participant based on every step taken during the running and walking trials. For statistical analysis, COPx and COPy ensemble averages for all participants were averaged into 10% intervals of stance.

Equation (1): Estimated COPy using loadsol data:
(1)

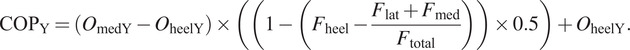

Equation (2): Estimated COPx using loadsol data:
(2)

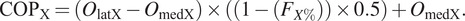




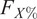
 , mediolateral force distribution; 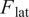 , lateral forefoot force; 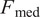 , medial forefoot force; 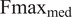 , peak medial forefoot force; 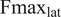 , peak lateral forefoot force; 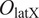 , lateral forefoot sensor’s mediolateral midpoint; 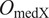 , medial forefoot sensor’s mediolateral midpoint; 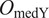 , medial forefoot sensor’s anteroposterior midpoint; 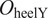 , heel sensor’s anteroposterior midpoint; 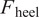 , heel force; and 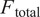 , sum of heel, medial forefoot, and lateral forefoot force.

### Statistical Analysis

Differences in COPx and COPy between pedar and loadsol in 10% intervals of stance were determined using limits of agreement (Bland and Altman, 1986). Additionally, the rank order of average COPx and COPy in 10% intervals of stance measured by pedar and loadsol was assessed using spearman’s correlation coefficient adjusted for multiple comparisons (*p* ≤ .003) (Curtin and Schulz, 1998).

## Results

**Figure 1.**
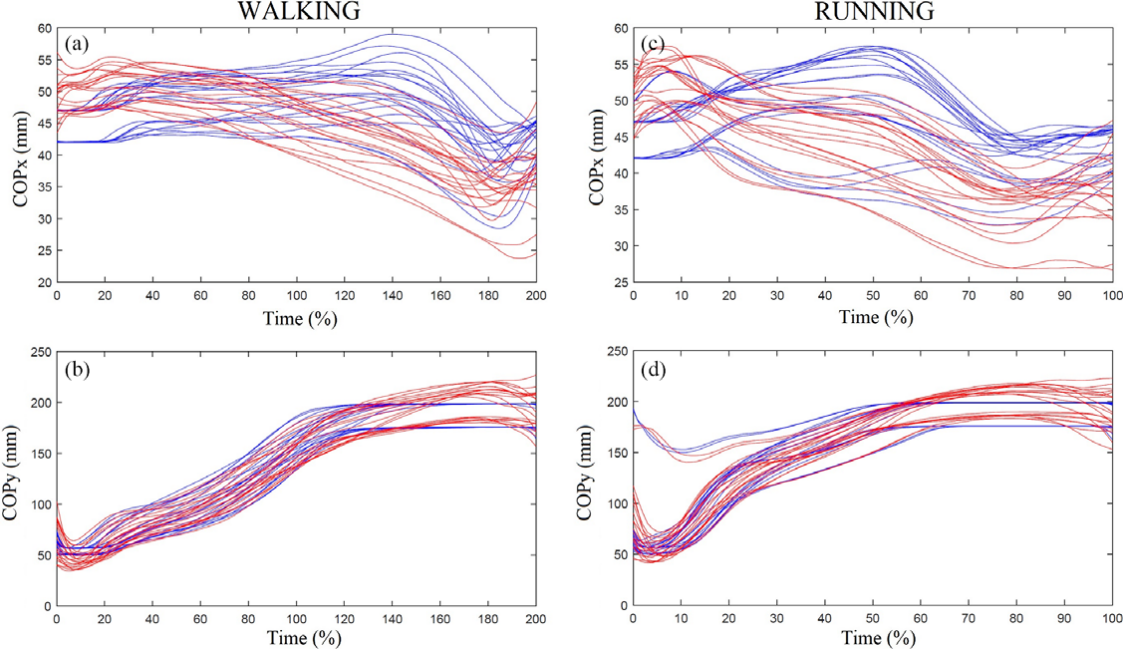
Ensemble average curves of pedar (red) and loadsol (blue) COPx (a) and COPy (b) during stance phase of walking and COPx (c) and COPy (d) during stance phase of running. For the COPx, larger numbers indicate lateral movement and for the COPy, larger numbers indicate anterior movement. There are four curves per participant in each graph as data were collected from two devices for two trials.

**Figure 2.**
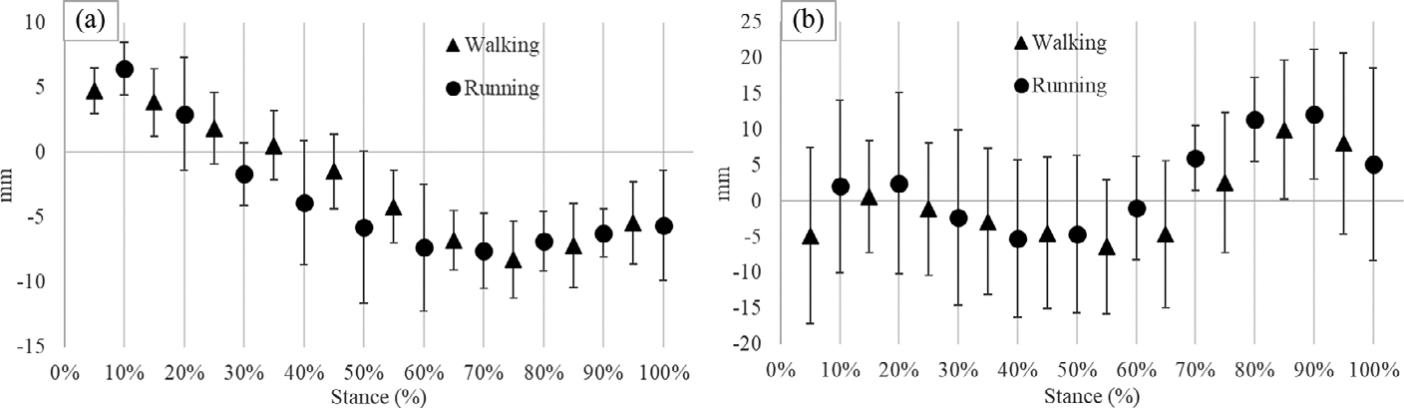
Average bias (shapes) and limits of agreement (error lines) between pedar and loadsol COPx (a) and COPy (b) during stance phase of walking and running. For COPx, a positive value indicates more lateral COPx in pedar, and a negative value indicates more medial COPx in pedar compared to loadsol. For COPy, a positive value indicates more anterior COPy in pedar, and negative value indicates more posterior COPy in pedar compared to loadsol.

Average self-selected speed was 1.3 ± 0.2 ms^−1^ for walking and 2.7 ± 0.5 ms^−1^ for running. Variability in self-selected walking and running speeds are accounted for in the within-subjects design of this study. One participant ran with a nonrearfoot strike pattern and was removed from the running analysis. Thus, data from nine participants were analyzed for running (Figure 1). Steps successfully recorded by both devices ranged from 20 to 68 during walking, and 59 to 100 during running. The rank order of COPx and COPy measured by pedar and loadsol had significant positive correlations for most of the stances during running and walking (Table 1). Average biases in the loadsol compared to the pedar ranged from −8.3 to 4.8 mm during walking and −7.6 to 6.5 mm during running in COPx, while average biases in COPy ranged from −6.4 to 10.0 mm during walking and −5.3 to 11.4 mm during running (Figure 2). Percent bias ranged from as low as 2 and 4% at 30% stance to as high as 6 and 17% at 80% stance relative to the loadsol COPy and COPx signals, respectively. The limits of agreement crossed 0 for both COPx and COPy only during the early stance phase of walking and running.

**Table 1.** Center of pressure averages from pedar and loadsol in mm during walking and running in 10% bins of stance phase with Spearman rank coefficients (*r*_s_) and *p*-value (*p*); means (standard deviation)

			Center of pressure (mm)			
Percentage of stance		Axis	Pedar	Loadsol	*r*_s_	*p*
10	Walking	*X* *	49.8 (2.8)	44.9 (2.8)	0.867	.001
		*Y* *	51.7 (9.8)	56.1 (5.1)	0.842	.002
	Running	*X*	51.6 (2.9)	45.2 (2.8)	0.700	.036
		*Y*	60.4 (9.9)	58.4 (5.7)	0.617	.077
20	Walking	*X*	50.8 (2.8)	46.9 (3.5)	0.794	.006
		*Y* *	71.7 (11.6)	70.7 (12.5)	0.855	.002
	Running	*X*	50.2 (4.0)	47.3 (3.3)	0.717	.03
		*Y*	90.2 (13.9)	87.7 (14.4)	0.817	.007
30	Walking	*X* *	50.1 (2.9)	48.2 (3.8)	0.952	<.001
		*Y* *	87.2 (11.8)	87.8 (14.2)	0.903	<.001
	Running	*X* *	47.3 (4.7)	49.0 (4.9)	0.917	.001
		*Y* *	126.4 (11.9)	128.8 (14.4)	0.900	.001
40	Walking	*X* *	49.0 (3.2)	48.4 (3.9)	0.939	<.001
		*Y* *	105.8 (13.7)	108.2 (16.5)	0.952	<.001
	Running	*X* *	45.6 (4.8)	49.5 (6.7)	0.933	<.001
		*Y* *	145.6 (11.0)	150.8 (13.7)	0.900	.001
50	Walking	*X* *	47.3 (3.7)	48.7 (3.9)	0.879	.001
		*Y* *	133.9 (14.0)	138.0 (16.6)	0.915	<.001
	Running	*X* *	44.4 (5.1)	50.1 (7.5)	1.000	<.001
		*Y* *	163.9 (11.6)	168.6 (14.4)	0.867	.002
60	Walking	*X* *	45.0 (4.1)	49.2 (4.2)	0.879	.001
		*Y*	162.7 (14.0)	168.7 (13.1)	0.782	.008
	Running	*X* *	42.3 (5.5)	49.7 (7.3)	0.933	<.001
		*Y* *	182.2 (11.8)	183.2 (14.0)	0.950	<.001
70	Walking	*X* *	42.9 (4.6)	49.7 (5.0)	0.976	<.001
		*Y* *	179.7 (13.8)	184.0 (11.2)	0.842	.002
	Running	*X* *	38.9 (5.1)	46.6 (5.8)	0.950	<.001
		*Y*	194.1 (12.5)	188.1 (12.4)	0.767	.016
80	Walking	*X* *	40.1 (4.5)	48.5 (5.9)	0.964	<.001
		*Y*	191.4 (14.4)	187.7 (12.1)	0.770	.009
	Running	*X*	36.0 (4.4)	42.8 (4.2)	0.850	.004
		*Y*	199.9 (14.0)	188.5 (12.2)	0.700	.036
90	Walking	*X* *	36.4 (4.3)	43.7 (5.7)	0.903	<.001
		*Y*	198.3 (15.9)	188.3 (12.2)	0.673	.033
	Running	*X* *	35.6 (3.9)	41.8 (3.5)	0.867	.002
		*Y*	200.6 (15.0)	188.5 (12.2)	0.833	.005
100	Walking	*X* *	35.5 (4.8)	40.9 (4.3)	0.842	.002
		*Y*	196.1 (16.5)	188.3 (12.2)	0.685	.029
	Running	*X* *	37.3 (4.7)	42.9 (3.1)	0.867	.002
		*Y* *	193.4 (15.5)	188.3 (12.4)	0.917	.001

* Denotes significance, *p* < .003.

## Discussion

The purpose of this study was to determine if the estimated COP from force data collected using a three-sensor loadsol was similar to COP measured using plantar pressure insoles during walking and running. While relative agreement between pedar and loadsol in COPx and COPy existed throughout the majority of the stance phase during walking and running, absolute agreement only existed in early stance when all sensors were loaded.

One potential application of COP estimates at early stance includes monitoring alterations in walking gait. The external peak knee adduction moment during walking occurs during early stance and may contribute to the development of knee osteoarthritis (Chehab et al., 2014). Frontal plane knee moments during walking are influenced by mediolateral alterations in COP (Haim et al., 2008). During the early stance phase of walking, a 14 mm lateral shift in COP significantly decreased the peak knee adduction moment in healthy adults (Haim et al., 2008). Based on our data, agreement from estimated COPx with loadsol was within 5.3 mm of COPx measured by pedar during the early stance phases of walking and running. Our equations may be applicable to monitoring lateral shifts in COP to alter knee loading during walking in the absence of motion capture or plantar pressure technology. Future studies should investigate the sensitivity of these equations to better understand the smallest detectable change in estimated COP for clinical application. Since the pedar system was limited to measuring COP at 200 Hz for only one insole at a time, the accuracy of estimating COP symmetry between limbs during walking and running using loadsol remains unknown and is another area for future exploration.

Some limitations of this study include the small sample size and low number of sensors in the loadsol insoles. As expected, the accuracy of our estimation of COP using the loadsol decreased when load was not applied to all sensors. Additionally, our estimation of COP using the loadsol was bounded by the midpoint of each sensor. In comparison, pedar insoles measure COP to the edges of the insole. Given these limitations, estimating COP with loadsol may yield more accurate results during standing assessments when all sensors are loaded throughout the entire trial. Moreover, accuracy of estimated COP is expected to increase with additional sensors per insole. In conclusion, force-sensing insoles with as little as three sensors were able to estimate COP location with good agreement during early stance when all sensors were loaded. However, agreement diminished near the beginning and end of stance phase during walking and running when some sensors were not loaded.

## Data Availability

The data that support the findings of this study are available from the corresponding author, C.M.B., upon reasonable request.
