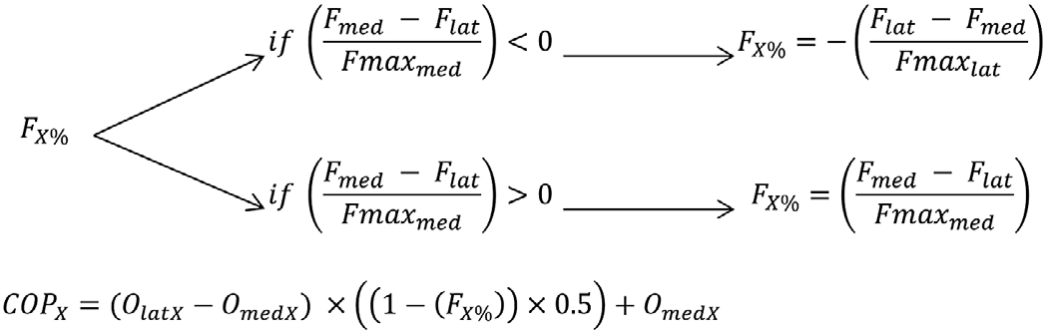# Validity of estimating center of pressure during walking and running with plantar load from a three-sensor wireless insole – ERRATUM

**DOI:** 10.1017/wtc.2023.22

**Published:** 2024-03-21

**Authors:** Richard A. Brindle, Chris M. Bleakley, Jeffrey B. Taylor, Robin M. Queen, Kevin R. Ford

Equation 2 was misprinted in the original article. The correct equation is listed below:

The Publisher apologises for the error.

**Figure:**